# The Role of Noncoding RNA in Airway Allergic Diseases through Regulation of T Cell Subsets

**DOI:** 10.1155/2022/6125698

**Published:** 2022-10-04

**Authors:** Shenghao Cheng, Qingping Tang, Shaobing Xie, Sihui Wen, Hua Zhang, Zhihai Xie, Weihong Jiang

**Affiliations:** ^1^Department of Otolaryngology-Head and Neck Surgery, Xiangya Hospital of Central South University, Changsha, Hunan, China 410008; ^2^Hunan Province Key Laboratory of Otolaryngology Critical Diseases, Changsha, Hunan, China 410008; ^3^National Clinical Research Center for Geriatric Disorders, Changsha, Hunan, China 410008; ^4^Department of Rehabilitation, Brain Hospital of Hunan Province, Hunan University of Chinese Medicine, Changsha, Hunan, China

## Abstract

Allergic rhinitis and asthma are common airway allergic diseases, the incidence of which has increased annually in recent years. The human body is frequently exposed to allergens and environmental irritants that trigger immune and inflammatory responses, resulting in altered gene expression. Mounting evidence suggested that epigenetic alterations were strongly associated with the progression and severity of allergic diseases. Noncoding RNAs (ncRNAs) are a class of transcribed RNA molecules that cannot be translated into polypeptides and consist of three major categories, microRNAs (miRNAs), long noncoding RNAs (lncRNAs), and circular RNAs (circRNAs). Previous studies showed that ncRNAs were involved in the physiopathological mechanisms of airway allergic diseases and contributed to their occurrence and development. This article reviews the current state of understanding of the role of noncoding RNAs in airway allergic diseases, highlights the limitations of recent studies, and outlines the prospects for further research to facilitate the clinical translation of noncoding RNAs as therapeutic targets and biomarkers.

## 1. Introduction

Airway allergic diseases, mainly asthma (AS) and allergic rhinitis (AR) are a group of chronic inflammatory diseases. Airway allergic diseases' main pathological features are the inflammatory response of the airway mucosa and airway tissue remodeling when individuals are exposed to airborne allergens, resulting in the involvement of multiple immune cells and the release of inflammatory mediators [1–3]. In recent years, the prevalence of allergic diseases has increased globally yearly, with the intensification of environmental pollution, which seriously adversely affects people's quality of life and learning [4, 5]. The occurrence of AS and AR results from a combination of factors, including individual differences, genetic inheritance, environmental exposure, and growth and development, all of which may be closely related to the onset of the disease. The key pathological features of both AS and AR, as heterogeneous chronic airway diseases, are recurrent inflammation, airway hyperresponsiveness, mucus hypersecretion, and reversible airway obstruction induced by the inflammatory cellular response [6–9].

**Figure 1:**
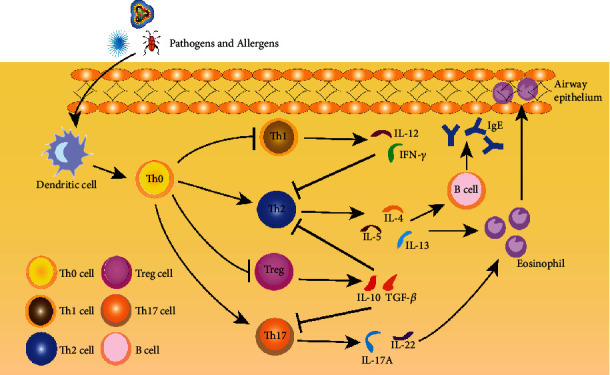
The interaction between innate and adaptive cells and type 2 inflammatory mediators underlies the pathophysiology of airway allergic disease. Disruption of the epithelium allows infiltration of viruses, bacteria, or allergens, activating innate and adaptive immune responses. Antigen presentation by dendritic cells activates the differentiation of naive T-helper cells (Th0 cell) to Th2 and Th17 cells and attenuates the differentiation to Th1 and Treg cells, immediately followed by the release of cytokines from Th2 and Th17 cells, leading to eosinophil recruitment, migration, and IgE production, and ultimately to the development of airway remodeling.

Researchers agreed that abnormal activation and function of intrinsic immune cells and adaptive immune cells (T helper 2 (Th2) cells) play an extremely critical role in the pathogenesis of airway allergic diseases [10–12]. Prior publication suggested that Th2 cells in the airway epithelium could produce various type 2 cytokines (IL-4, IL-5, and IL-13), which in turn promote eosinophil recruitment, while these cytokines play a key role in airway epithelial cell activation, chemoattraction of effector cells, regulation of airway smooth muscle, and remodeling of the epithelial matrix [13, 14]. In addition, the balance between Th17 cells and T regulatory cell (Tregs) cells is similarly thought to be associated with developing airway allergic diseases [15, 16]. Thus, tapping into the regulatory mechanisms of innate and adaptive immune cells from different perspectives is currently a hot spot and frontier in airway allergic disease research [17, 18] (Figure 1).

Th0 cell, T helper 0 cell; Th1 cell, T helper 1 cell; Th2 cell, T helper 2 cell; Treg cell, T regulatory cell; Th17 cell, T helper 17 cell; IL-4, Interleukin-4; IL-5, Interleukin-5; IL-13, Interleukin-13; IL-10, Interleukin-10; IL-12, Interleukin-12; IL-17A, Interleukin-17A; IFN-*γ*, Interferon- gamma; TGF-*β*, Transforming growth factor-beta.

**Figure 2:**
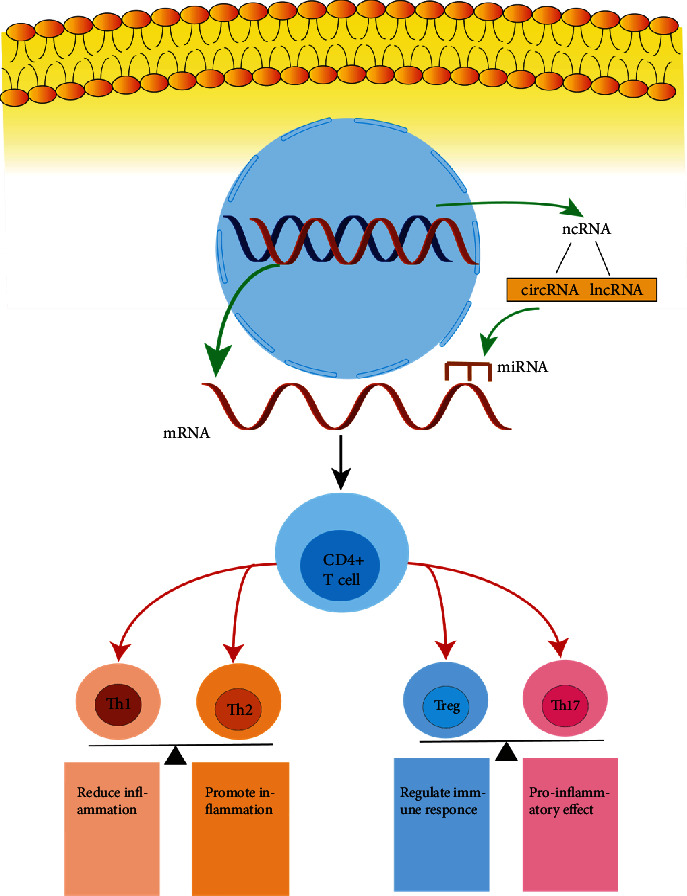
ncRNA regulates the mechanism of CD4^+^ T cell differentiation. ncRNA affects miRNA level via molecular sponge action, which can influence CD4^+^ T cell differentiation by binding to mRNA encoding CD4^+^ T cell genes, resulting in an imbalance between Th1 and Th2, Th17 and Treg. Thereby, exacerbating or reducing airway remodeling, inflammatory mediators release, and inflammatory responses.

In recent years, noncoding RNAs (ncRNAs), mainly miRNA, lncRNA, and circRNA, have been found to have a significant relationship with the occurrence and development of airway allergic diseases. [19, 20] Therefore, a deeper exploration of the role of ncRNAs in airway degeneration and related regulatory mechanisms is expected to provide new directions for the investigation of biomarkers for diagnosis, treatment, and prediction of disease prognosis. This review summarizes the role of ncRNAs in airway allergic diseases and investigates their regulatory mechanisms on T cells and their effects on downstream cytokines to better understand the pathogenesis of airway allergic diseases. (Figure 2).

ncRNA, noncoding RNA; miRNA, microRNA; lncRNA, long noncoding RNA; circRNA, circular RNA; Th1 cell, T helper 1 cell; Th2 cell, T helper 2 cell; Treg cell, T regulatory cell; Th17 cell, T helper 17 cell.

## 2. ncRNA and AS

### 2.1. miRNA and AS

Increasing attention has been paid to the linkage of epigenetic modifications in AS pathology and a series of results have been obtained. miRNAs, consisting of 22-24 single-stranded nucleotides, are an essential component of epigenetic regulation with crucial regulatory roles in immune cells [21, 22]. miRNA functions primarily as a repressor of gene expression at the posttranscriptional level by binding to complementary sequences in the target mRNA and without altering the genomic sequence [22–26]. Previous studies confirmed that miRNAs play an essential role in allergic diseases by influencing Th1/Th2 polarization and Tregs cell/Th17 cell imbalance, promoting epithelial chronic inflammation and tissue remodeling, and activating intrinsic immune cells [11, 27, 28]. Recently, researchers screened and validated various miRNAs that affected the development of AS by regulating immune cell function and promoting the release of inflammatory mediators [29–31]. Mattes et al. [32] reported that airway hyperactivity and inflammation might be reduced by inhibiting miR-126 expression, which could affect CD4^+^ T cell differentiation towards Th2 and the release of inflammatory cytokines. As important inflammatory factors, interleukin-33 (IL-33) and IL-13 could activate Th2 cells, mast cells, dendritic cells, eosinophils, and basophils, which promote the development of AS disease [33, 34]. Thus, screening for miRNAs can bind to IL-33 or IL-13 mRNA, which inhibit the expression of IL-33 or IL-13, and further exploring the potential regulatory mechanisms would help alleviate the disease progression of AS. A recent study found that miR-200b and miR-200c were downregulated in alveolar lavage fluid-derived cells from AS patients and demonstrated their ability to bind to the 3′ nontranscribed region (UTR) of IL-33 mRNA and thus affect the expression level of IL-33 by in vitro and in vivo experiments [35]. In addition, the miRNA-let-7a family was shown to target the IL-13 mRNA, resulting in lower levels of IL-13 and alleviating airway inflammation [36]. Notably, matrix metallopeptidase-16 (MMP-16) can play an essential role in tissue remodeling and airway inflammation by activating proMMP-2 [37–40]. Lou et al. [41] showed that miR-192-5p plays an inhibitory role in airway remodeling and autophagy reduction in asthma patients by targeting MMP-16 and autophagy-related protein 7 (ATG7). In addition, phosphatase and tensin homolog (PTEN), and MAPK/STAT1 pathway are critical regulatory pathways in allergic diseases [42]. It was shown that overexpression of miR-19a in the airway enhanced Th2 cytokine production and reduced miR-19a levels in airway smooth muscle cells, which could promote airway remodeling by directly targeting PTEN and MAPK/STAT1 signaling pathways [43, 44]. Besides, a study by Zhang and colleagues [45] found that decreasing miR-221-3p expression in epithelial cells could reduce inflammation by upregulating anti-inflammatory chemokine ligand 17 (CXCL17), which in turn inhibited the expression of chemokine c-c motif ligand 24 (CCL24), CCL26 and osteochondral proteins because these cytokines act as a key role in the recruitment of eosinophils and macrophages to the airway [45–48]. Recently, there were also findings that miRNAs transported by extracellular vesicles of serum and immune cell origin could mediate intercellular communication and play a significant role in the development of AS by regulating immune cells [49–51]. Li et al. [52] found that macrophage-derived exosome transporting miR-21-5p could promote epithelial-mesenchymal transition of airway mucosal epithelial cells by targeting Smad7, consequently exacerbating airway inflammation and airway stenosis. In another study, researchers found that adipose mesenchymal stem cell-derived exosomal delivery miR-301a-3p targets the STAT3 pathway to regulate the involvement of airway smooth muscle cells in the disease development of AS. [53] Based on the above findings, miRNAs may be involved in the development and progression of AS by affecting intrinsic and adaptive immune functions and regulating the release of various inflammatory mediators and activating signaling pathways. These specific miRNAs may be used as therapeutic targets for AS. Additional miRNAs associated with AS are described in detail in Table 1.

### 2.2. lncRNA and AS

lncRNAs are composed of more than 200 nucleotides with tissue and cellular specificity, and their functions include epigenetic regulation and induction of immune cell differentiation [22]. lncRNAs could facilitate or attenuate the translation of target mRNAs and even alter the stability of mRNAs and proteins through three main pathways: (1) acting as regulators of genomic transcription in the nucleus; (2) participating in posttranscriptional regulation in the cytoplasm; (3) secreting exosomes or other means to the outside of the cell and participating in cross-cellular talks [54–59]. lncRNAs were proven to play an integral role in the pathogenesis of AS by regulating the differentiation and apoptosis of hematopoietic stem cells, bone marrow cells, and the activation of monocytes, macrophages, and dendritic cells in immune regulation [60]. Previous studies demonstrated that lncRNAs could unlock the binding of miRNAs to the 3′ UTR of target genes by binding miRNAs as molecular sponges and then regulating the mRNA transcription of target genes in immune cells, ultimately affecting the release of inflammatory mediators and immune response [61]. Qiu et al. [62] found that lncRNA-MEG3 could act as competitive endogenous RNA to regulate the Tregs/Th17 balance in asthma patients by targeting miRNA-17, which could contribute to Th17 cell differentiation and affect disease progression. Additionally, Liang and Tang [63] found that lncRNA-MALAT1 could compete with miRNA-155 and subsequently alter the Th1/Th2 balance within CD4^+^ T cells, impacting Th2 cytokine levels and the development of asthma. The nuclear factor-*κ*B (NF-*κ*B) signaling pathway, an essential signaling regulatory pathway, affects the transcription of proinflammatory cytokines such as interleukin-1*β* (IL-1*β*), tumor necrosis factor-*α* (TNF-*α*), and interleukin-6 (IL-6), all of which are closely associated with the development of AS [64, 65]. Moreover, increasing numbers of investigators are finding that multiple lncRNAs can be used as objective biomarkers for AS diagnosis, disease severity and prognosis assessment. Feng et al. [66] found that lncRNA-MEG3 was highly expressed in the serum of AS patients, and its elevated levels were correlated with the different inflammatory types and courses of AS. Xu et al. [67] found that lncRNA PCGEM1 could enhance the anti-inflammatory and respiratory protective effects of montelukast sodium in children with cough variant AS by blocking the activation of the NF-*κ*B signaling pathway. In another study, significant variability in lncRNA expression profiles was found, and lncPVT1 was tested as a predictor of the occurrence of airway remodeling in AS patients by collecting smooth muscle cells of airway origin from AS patients and normal controls for transcriptome sequencing [68]. A recent study found that the lncRNA-ANRIL/miR-125a axis was upregulated and positively correlated with disease severity in plasma samples collected from patients of varying severity, healthy subjects, and patients with worsening bronchial AS [69]. In another study, lncRNA GAS5 was identified as a potential biomarker for the early diagnosis of severe AS [70]. These studies suggested that lncRNAs were not only involved in the development of AS but that their expression levels could be closely related to the clinical severity of the disease. Importantly, exosome-carried lncRNAs have also been shown to be involved in the development of AS [71, 72]. Zhang et al. [73] found that activated neutrophil-derived exosomes transporting the lncRNA CRNDE effectively promote differentiation and migration of airway smooth muscle cells, which were closely associated with disease progression and airway remodeling in AS. Other lncRNAs associated with AS disease are detailed in Table 1. Therefore, it is expected that new ideas for the precise treatment of AS can be provided by targeting and regulating specific lncRNAs and downstream signaling pathways, and the related molecular mechanisms are yet to be further explored in-depth.

### 2.3. circRNA and AS

CircRNA is a newly discovered endogenously expressed ncRNA characterized by a loop structure without 5′-3′ polarity and a polyphyletic acid tail [74–76]. CircRNA has been shown to be involved in pathophysiological processes in various diseases, such as diabetes, cardiovascular diseases, neurological diseases, and tumors [77–81], and can similarly act as miRNA sponges to regulate gene expression [82, 83]. Several studies have found that circRNAs could be involved in developing AS by regulating innate and adaptive immune responses in recent years [22, 84]. A recent study found that hsa_circ_0005519 could regulate the expression of IL-6 and IL-13 in CD4^+^ T cells by targeting hsa-let-7a-5p, which influenced the development of AS [82]. In another study, circHIPK3 was shown to influence the pathological process of AS by regulating the miR-326/STIM1 axis regulating the proliferation of airway smooth muscle cells [85]. In particular, circRNA levels were found to be a potential objective assessment marker for diagnosing AS and disease severity [86]. Huang et al. [86] found that upregulation of hsa_circ_0002594 was positively correlated with exhaled nitric oxide levels, and its expression was positively correlated with the patient's family history, positive skin prick test (SPT), and Th2 cytokine expression levels. To date, only a few circRNA mechanisms of action in AS have been initially explored (Table 1), and there are no studies on the expression profile and mechanisms of exosomal-derived circRNAs in pathological specimens from AS patients.

## 3. ncRNA and AR

### 3.1. miRNA and AR

Although some scholars have observed some similarities between AR and AS in terms of disease onset and immune response and proposed the concept of “one airway, one disease”, significant differences still exist in the pathological mechanisms and targets of intervention between the two diseases. Moreover, differentially expressed miRNAs could be involved in the development of AR by affecting the function of innate and adaptive immune cells and the level of inflammatory mediators [124–126]. A previous study found that modulation of miRNA-let-7e and miR-let-7 overlap could effectively regulate the expression levels of various inflammatory factors in AR mouse models and nasal mucosal epithelial cell models [36, 127]. In addition, Gao and Yu [128] found that miRNA-16 inhibited IL-13-induced inflammatory cytokine secretion and mucus production in nasal epithelial cells by suppressing the I*κ*B kinase *β*/NF-*κ*B pathway, which could promote Th2 cell differentiation. Recent studies have identified multiple miRNAs that could be involved in PM2.5-induced AR inflammation by inhibiting autophagy and regulating the AKT/mTOR pathway, which could prompt Treg/Th17 cell imbalance [124, 125]. In addition, various miRNAs were confirmed to be correlated with the diagnosis, disease severity, and treatment efficacy of AR [129]. Previous studies reported that serum miRNA-223 levels in AR patients were higher than normal controls and positively correlated with serum eosinophil cationic protein, eosinophil count, and total nasal symptom score (TNSS), suggesting that miRNA-223 has been involved in AR eosinophilic inflammation and disease progression [130, 131]. Interestingly, miRNA expression profiles were associated with the efficacy of AR-specific immunotherapy, where patients received treatment with significant changes in multiple miRNA expression levels [132, 133]. Other miRNAs associated with AR disease are detailed in Table 2. In conclusion, miRNAs can be involved in AR pathogenesis by regulating immune cell activity and releasing inflammatory factors. Further exploration of their potential mechanisms could provide a theoretical basis for future precision treatment of AR.

### 3.2. lncRNA and AR

Many previous studies confirmed that lncRNAs have a variety of important biological activities, including DNA damage, programmed cell death, development, inflammation, tumorigenesis, and immune response [134, 135]. In recent years, researchers focused on the differential expression levels of lncRNAs in nasal mucosal tissues of AR patients and mouse models and their involvement in disease development by affecting different downstream signaling pathways [134, 136, 137]. Yue et al. [138] demonstrated that lncRNA00632 inhibited Th2 cell differentiation and IL-13 release by adsorbing miRNA-498, indicating a protective role of lnc00632 in AR. The JAK signaling pathway is a critical cytokine signaling pathway [139, 140]. In contrast, the Th2-associated cytokines IL-4, 5, and 13 are associated with activating the JAK2 and STAT6 signaling pathways, respectively [141, 142]. Liu et al. [143] identified lncANRIL as a potential new target for the treatment of AR by knocking down lncANRIL to modulate the miR-15a-5p/JAK2 signaling axis and consequently inhibit the secretion of IL-13. Moreover, the literature has reported that lncRNA expression profiles in immune cells of AR patients and animal models are equally cell-specific [144, 145]. Ma et al. [146] found that the expression profiles of lncRNAs were significantly cell-specific and involved multiple signaling pathways associated with AR disease development by comparing the expression profiles of lncRNAs in CD4^+^ T cells from AR mouse models and control mice by sequencing. In parallel, some lncRNAs have been proven to be potential biomarkers for assessing AR severity and prognosis. In a recent study, histopathological specimens revealed that lncRNA-NEAT1 expression was significantly upregulated in the nasal mucosa of AR patients and positively correlated with disease symptom scores and inflammatory cytokine levels, suggesting that it could be used as a biomarker to assess the severity of AR disease [140]. Moreover, a recent study found that circulating-derived lncRNAs also play an essential role in the pathogenesis of AR [147–149]. Wang et al. [148] found that the exosome-derived lncRNA NEAT1 regulates the microRNA-511/NR4A2 signaling axis and then participates in the disease development of AR. The above studies suggested that both nasal mucosal and circulating sources of lncRNAs could be involved in developing AR disease through different pathways. The potential regulatory mechanisms need to be explored in further studies. Additional lncRNAs associated with AR disease are detailed in Table 2.

### 3.3. circRNA and AR

circRNA, an emerging endogenous ncRNA, also plays a critical role in the immune and inflammatory responses [150, 151]. Chen et al. [152] identified circRNA expression profiles in the nasal mucosa of AR mice using RNA sequencing and found 51 circRNAs upregulated and 35 circRNAs downregulated, with some circRNAs involved in activating T and B cells. In another study, investigators analyzed circRNAs in the nasal mucosa of AR patients and controls using high-throughput sequencing. They explored the possible role and mechanism of the circRNA-miRNA-mRNA interaction network in AR pathology by bioinformatic analysis [153]. A previous study confirmed that GATA3 plays a crucial role in developing Th2 cells and two innate lymphocytes [154], whose signaling is a key process inducing Th2 cell development [155, 156]. GATA3 could induce chromatin remodeling at Th2-related loci and enhance Th2 cytokine production [157]. A new study revealed that circHIPK3 was highly expressed in the nasal mucosa of AR mice, and it acted as a sponge for miR-495 and deregulated the transcriptional repression of GATA3, promoting CD4^+^ T cells to Th2 and secreting cytokines that exacerbate d ovalbumin-induced nasal symptoms [158]. Investigators identified an essential regulatory role for circARRDC3/miR-375/KLF4z in developing IL-13-induced inflammation in nasal mucosal epithelial cells by accelerating Th2 differentiation [159]. Currently, studies on the role and mechanism of circRNA in AR are less circRNA expression in AR nasal mucosa and peripheral blood. The related mechanism of action remains to be further explored.

## 4. Conclusion and Perspective

As the most common airway allergic diseases, AS and AR seriously affect patients' quality of life and impose a substantial economic burden on society. Therefore, it is of great clinical value to explore their pathogenesis and treatment precisely. In recent years, ncRNAs have been used as a new biomarker for disease treatment research, especially lncRNAs and circRNAs are the current hot spots in epigenetic research. However, circRNAs have been relatively poorly explored in AS and AR. In this review, most miRNAs, lncRNAs, and circRNAs currently have essential roles in developing AS and AR from three initial aspects, respectively. miRNAs can participate in the pathogenesis of AS and AR by targeting target genes to inhibit their expression in innate and adaptive immune cells. At the same time, lncRNAs and circRNAs are mainly involved in the development and progression of AS and AR by binding to the corresponding miRNAs through the ceRNA mechanism to relieve the inhibitory effect of the latter on target genes and regulate immune cells through downstream signaling pathways. The role of circulating ncRNAs, especially exosomal-transported ncRNAs, is gradually coming into the view of researchers in AS and AR, and whether they can be used as objective biomarkers for diagnosis, disease symptom assessment, and prognosis prediction is still under investigation. Follow-up studies should explore the role and mechanism of ncRNAs in the development and progression of AS and AR from multiple perspectives to provide new ideas for future diagnosis, treatment, and prognosis of the diseases.

## Figures and Tables

**Table 1:** The expression and mechanisms of ncRNAs in asthma.

ncRNA	Expression level	Signaling pathways or targets	Function
miRNA-1248	Upregulation	Unknown	Elevate Th2 cytokine levels [87]
miRNA-126	Upregulation	DNMT1	Promote inflammation [88]
miRNA-21	Upregulation	PI3K/Akt, IL13R*α*1, STAT6	Modulate ASMCs proliferation, migration, and modulate IL-12 [89, 90]
miRNA-21	Upregulation	STAT4	Decrease IL-12 levels [90]
miRNA-98	Upregulation	Unknown	Suppress the expression of TSP1 [91]
miRNA-155	Upregulation	PGE2	Enhance COX2 expression [92]
miR-371 miR-138 miR-544 miR-145 miR-214	Upregulation	Runx3	Balance Th1/Th2 [93]
miRNA-16	Upregulation	ADRB2	Predictive biomarker of therapeutic response [94]
miRNA-146a-5p	Upregulation	5-LO	Attenuate inflammation [95]
miRNA-30a	Upregulation	ATG5	Decrease inflammation [96]
miRNA-126	Downregulation	GATA3	Diminish Th2 response [32]
miRNA-200	Downregulation	Unknown	Inhibit IL-33 levels [35]
miRNA-let-7a	Upregulation	Unknown	Decrease IL-33 levels [36]
miR-192-5p	Upregulation	MMP-16, ATG7	Enhance airway remodeling and autophagy [41]
miR-19a	Upregulation	PTEN, MAPK/STAT1	Enhance airway remodeling and Th2 [43, 44]
miR-221-3p	Upregulation	CXCL17	Aggravate inflammation [45]
miRNA-221	Downregulation	Unknown	Reduce airway inflammation [97]
miR-142-3p	Downregulation	WNT	Regulate proliferation and differentiation of ASMCs [98]
miRNA-34a	Downregulation	FoxP3	Attenuate inflammation [99]
miRNA-410	Downregulation	Unknown	Decrease IL-4/IL13levels [100]
miR-218-5p	Downregulation	CTNND2	Suppress chemokine expression [101]
miRNA-192	Downregulation	CXCR5	Suppresses T helper cell [102]
miRNA-485	Downregulation	TGF-*β*/Smads	Decrease smurf2 levels [103]
miR-21-5p	Downregulation	Smad7	Promote epithelial-mesenchymal transition [52]
miR-301a-3p	Downregulation	STAT3	Activate smooth muscle cells [53]
lncRNA-MEG3	Upregulation	miRNA-17/ ROR*γ*t	Regulate Treg/Th17 balance [62]
lncRNA-MALAT1	Upregulation	miRNA-155	Promote Th2 inflammation [63]
lncRNA PCGEM1	Upregulation	NF-*κ*B	Ameliorate inflammation [67]
lncRNA CRNDE	Upregulation	Unknown	Enhance airway remodeling [73]
lncRNA-BAZ2B	Upregulation	Unknown	Promote M2 macrophage activation [104]
lncRNA-000127	Upregulation	Unknown	Promote Th2 inflammation [105]
lncRNA-TCF7	Upregulation	TIMMDC1/Akt	Promote the growth and migration of ASMCs [106]
lncRNA-PVT1	Upregulation	miRNA-149, miR-15a-5p, miR-29c-3p	Exacerbate inflammation and impact Th1/Th2 imbalance [107, 108]
lncRNA-PVT1	Upregulation	miR-590-5p/FSTL1	Attenuate airway remodeling [68, 109]
lncRNA-ANRIL	Upregulation	miRNA-125a	Exacerbate severity and inflammation [69]
lncRNA-Malat1	Upregulation	miR-150-eIF4E/Akt	Exacerbate inflammation [110]
lncRNA-NEAT1	Upregulation	microRNA-124	Increase inflammation [111]
lncRNA- n337374	Upregulation	CD86 and ERK	Ameliorate inflammation [112]
lncRNA-BCYRN1	Upregulation	Receptor potential 1	Promote inflammation [113]
lncRNA-TUG1	Upregulation	microRNA181b/HMGB1	Promote inflammation [114, 115]
lncRNA- LASI	Upregulation	MUC5AC	Promote inflammation [115]
lncRNA-H19	Downregulation	PI3K/Akt/NF-kB, miR21/PTEN/Akt	Attenuate inflammation [116, 117]
lncRNA-AK169641	Downregulation	Unknown	Increase eosinophils infiltration [118]
lncRNA-TUG1	Downregulation	miR-29c/B7-H3	Promote Th2 cell differentiation [20]
lncRNA-AK085-865	Downregulation	Unknown	Ameliorate inflammation [119]
lncRNA-BCYRN1	Downregulation	miRNA-150	Inhibit the proliferation of ASMCs [113]
lncRNA-LINCPINT	Downregulation	miRNA-265p/PTEN	Retard the abnormal growth of ASMCs [120]
circRNA-0005519	Upregulation	miRNA-7a-5p	Increase IL-6/IL-13levels [82]
circRNA-HIPK3	Upregulation	miR-326/STIM1; miR-375/MMP-16	Modulate the proliferation of AMSCs [85, 121]
circRNA-0002594	Upregulation	Unknown	Upregulate in CD4^+^ T cells [86]
CircRNA-ZNF652	Upregulation	miR-452-5p/JAK2	Promote the goblet cell metaplasia [122]
circRNA-ERBB2	Downregulation	miR-98-5p/IGF1R	Increase infiltration [123]

**Table 2:** The expression and mechanisms of ncRNAs in AR.

ncRNA	Expression level	Signaling pathways or target	Function
miRNA-223	Upregulation	Unknown	Promote inflammation [130]
miRNA-155	Upregulation	Unknown	Regulate Th2 factors [160]
miRNA-202-5P	Upregulation	MATN2	Promote M2 polarization [161]
miRNA-202-5p	Upregulation	MATN2	Promote Tregs polarization [162]
miRNA-17-5P	Upregulation	ABCA1/CD69	Aggravate seasonal AR [163]
miRNA-375	Upregulation	JAK2/STAT3	Ameliorate AR [164]
miRNA-223-3p	Upregulation	INPP4A	Enhance eosinophil infiltration [165]
miRNA-let-7a	Upregulation	OPEN	Regulate Th2 cells [166]
miRNA-17-92	Upregulation	Unknown	Exacerbate AR Inflammation [167]
miRNA-15a-5p	Downregulation	ADRB2	Inhibit IL-13 levels [168]
miRNA-155	Downregulation	SOCS1and SIRT1	Promote Tregs differentiation [169]
miRNA-181a	Downregulation	PI3K/AKT	Upregulate IL-10 and TGF-*β* [169]
miRNA-146a	Downregulation	TLR4/TRAF6/NF-*κ*B	Regulate Th2 cells [170]
miRNA-466a-3p	Downregulation	GATA3	Attenuate inflammation [171]
miRNA-345-5p	Downregulation	TLR4/NF-*κ*B	Increase anti-inflammatory factors [172]
miRNA-29	Downregulation	CD276	Reduce IL-4, IL-6 level [173]
miRNA-133b	Downregulation	Nlrp3	Ameliorate allergic inflammation [174]
miRNA-106b	Downregulation	Egr-2	Regulate Th2 polarisation [175]
miRNA-143	Downregulation	IL13Ra1	Inhibit inflammation [176]
miRNA-30a-5p	Downregulation	SOCS3	Involved in AR pathogenesis [177]
miRNA-135a	Downregulation	Unknown	Regulate Th1/Th2 imbalance [11]
miRNA-let-7e	Downregulation	SOCS4	Anti-inflammatory [127, 128]
miRNA-16	Downregulation	I*κ*B kinase *β*/NF-*κ*B	Inhibit IL-13 secretion [128]
miRNA-487b	Downregulation	IL-33/ST2	Inhibit IL-13 secretion [178]
lncRNA SNHG16	Upregulation	miR-106b-5p/JAK1/STAT3	Promote inflammation [179]
lncRNAGABPA-9 : 1, NR103763, CCL21, APOA2, RAD9B-1 : 4	Upregulation	Unknown	Involved in AR pathogenesis [134]
lncRNA-ANRIL	Upregulation	miR-15a-5p/JAK2	Suppress inflammation [143]
lncRNA-NEAT1	Upregulation	miR-21, miR-125a	Affect allergy inflammation [180]
lncRNA-GAS-5	Downregulation	EZH2 and T-bet	Promote Th2 differentiation [181]
lncRNA-GAS-5	Downregulation	miR-21 and miR-140	Affect Th1/Th2 imbalance [182]
lncRNAFOXD3-AS1	Downregulation	Unknown	Inhibit Th2 immunoreaction [183]
LncRNATCONS_00147848	Downregulation	JAK/STAT3	Reduce inflammatory response [184]
lncRNA-000632	Downregulation	miRNA-498	Inhibit IL-13[143]
circRNA-HIPK3	Upregulation	miRNA-495	Promote Th2 differentiation [158]
circRNA-ARRDC3	Downregulation	miR-375/KLF4	Promote inflammatory [185]
circRNA-0000520	Downregulation	miR-556-5p/NLRP3	Attenuate inflammatory [186]
